# Cross-cultural adaptation and validation of the Chinese version of the Perceived Emotional Expression Scale for adolescents

**DOI:** 10.3389/fpsyt.2025.1680435

**Published:** 2025-12-09

**Authors:** Jianing Duan, Youbei Lin, Chuang Li, Wangxiao Zhao, Xiuli Wang

**Affiliations:** 1 Oncology Clinical Research Ward, The First Affiliated Hospital of Jinzhou Medical University, Jinzhou, Liaoning, China; 2 School of Nursing, Jinzhou Medical University, Jinzhou, China

**Keywords:** perceived expressed emotion, general adolescent, non-psychiatric population, validity, cross-cultural adaptation

## Abstract

**Background:**

Expressed emotion, as a crucial indicator of the family environment, has been proven to be a highly accurate psychosocial predictor of psychiatric relapse. At present, there is no standardized assessment tool to evaluate family-expressed emotions from the perspective of adolescents. This study aims to introduce the PEES-GAP scale into Chinese and assess its reliability and validity among general adolescent populations in China.

**Methods:**

This study first used a modified Brislin model to translate the PEES-GAP scale into Chinese. Subsequently, six experts in related fields were invited to assess the Item-level content validity index (I-CVI) of the translated scale. Using convenience sampling, 827 adolescents meeting the inclusion criteria were recruited from two secondary schools and two universities in Shandong Province and Liaoning Province, China. The reliability of the scale was assessed through internal consistency, split-half reliability, and test-retest reliability. To examine the construct validity of the Chinese version of the PEES-GAP, exploratory factor analysis (EFA) was first conducted, followed by confirmatory factor analysis (CFA) to further assess its construct validity.

**Results:**

Based on retaining the original 19 items of the scale, the final C-PEES-GAP demonstrated excellent psychometric properties, with a Cronbach’s alpha coefficient of 0.928. The I-CVI for each subscale ranged from 0.830 to 1.000, with split-half reliability of 0.855 and test-retest reliability of 0.964. The three-factor exploratory factor model explained 59.295% of the total variance, indicating a robust factor structure. The fit indices obtained from CFA included: CMIN/DF = 2.791, RMSEA = 0.077, AGFI = 0.832, TLI = 0.924, IFI = 0.935, CFI = 0.934, GFI = 0.869, and PGFI = 0.677.

**Conclusion:**

Following rigorous translation and validation procedures, the PEES-GAP has been adapted for use in China, demonstrating robust psychometric properties. It has emerged as a valid tool for assessing perceived family emotional expression among the general adolescent population in China. Furthermore, this scale may also serve as a crucial instrument for researchers in the fields of psychology and mental health to conduct relevant research, as well as for clinical professionals to develop targeted intervention strategies addressing adolescents’ emotional issues.

## Background

Adolescence is a critical period for emotional development. During this developmental stage, adolescents who fail to successfully adjust their emotional responses to adapt to the surrounding environment may face emotional problems (1), including anxiety, depression, and fear. Families often play a vital role during this period. Without proper guidance from family members and the influence of a positive family atmosphere, a series of adverse consequences may occur, such as declining academic performance, sleep disorders, unintended pregnancy, and even suicide (2). For these reasons, the expressed emotion of parents and guardians plays an important role in adolescence.

Expressed emotion refers to the emotional attitudes of caregivers toward family members with mental illnesses (3). It serves as a benchmark for assessing the family environment, precisely measuring three to five dimensions of such an environment—most notably critical comments, hostility, and emotional over-involvement (4). High expressed emotion (HEE) has been identified as a risk factor that worsens mental illness and prolongs its duration (5).

Research indicates that bad family-expressed emotion poses a threat to the mental and physical well-being of adolescents in the general population (6). A study on emotional abuse in the Iranian population revealed that among adolescents, the perception of low negative expressed emotion within the family (e.g., less criticism, no obvious hostility) was negatively correlated with emotional dysregulation caused by childhood trauma, particularly emotional abuse (7). This kind of perception can create a psychologically safe environment for emotional expression, reduce the consumption of regulatory resources, and alleviate psychological distress. In contrast, the perception of high negative expressed emotion (e.g., frequent criticism, obvious hostility) reinforces the subjective experience that one’s emotional expression is not accepted, and exacerbates the impairment of emotional processing functions caused by trauma. This underscores the need for adolescents to objectively assess the emotions directed at them from their own perspective.

However, due to a lack of attention to and consideration for non-clinical adolescent populations, such assessment tools are currently unavailable (8). Existing instruments for evaluating expressed emotion include Magana et al.’s Five-Minute Speech Sample (FMSS) for patients’ relatives (9), Vaughn & Leff’s Camberwell Family Interview (CFI) (10), and Cole et al.’s Level of Expressed Emotion Scale (11). The aforementioned measurement tool only assesses expressed emotions from the perspective of family members, ignoring the adolescents’ own feelings. Moreover, it is only applicable to evaluating the families of patients and cannot be applied to the families of non-clinical adolescents.

To address this gap, Professor Morenikeji Fausiat Hamzat’s team developed the Perceived Expressed Emotion Scale for the General Adolescent Population (PEES-GAP) through systematic literature reviews and empirical research (12), following rigorous scale development protocols. This scale aims to overcome the limitations of traditional tools by systematically synthesizing research on adolescents’ family emotional expression and identifying core elements closely linked to adolescents’ psychological development and family interactions. Unlike other adolescent-focused expressed emotion scales, PEES-GAP is enhanced in psychometric properties: it is culturally sensitive to ensure relevance across diverse backgrounds and includes both positive (e.g., warmth, support) and negative (e.g., criticism, hostility) emotional interactions. It demonstrates high internal consistency and test-retest reliability, with extensive validation to ensure accuracy in measuring expressed emotion from adolescents’ perspectives. Additionally, it is user-friendly and inclusive, making it suitable for both clinical and non-clinical adolescent populations. The scale covers dimensions such as perceived critical comments, hostile atmospheres, and emotional over-involvement.

This study takes Chinese adolescent groups as the specific target and focuses on the field of psychometrics. By strictly following psychometric research norms and applying scientific research design, data collection, and analysis methods, it aims to construct and validate a measurement tool for Chinese adolescents’ perceived expressed emotion.

## Methods

### Ethical consideration

This study was approved by the Ethics Committee of Jinzhou Medical University (JZMULL2025300), and all research procedures complied with the ethical guidelines of the committee. Informed consent was obtained from all participants before data collection.

### Sampling procedure

This study was conducted from April 1, 2025 to July 1, 2025. Convenience sampling was used to recruit adolescent volunteers from two middle schools and two universities in Qingdao City and Jinzhou City, China. Inclusion criteria include: I. Adolescents aged 13-19; II. Adolescents with certain cognitive ability and understanding level, who can understand the text descriptions and the meaning of questions in the scale; III. Voluntary participation in this research. Exclusion criteria are: I. Adolescents who have experienced major life events (such as the death of a relative, parental divorce, etc.) within 3 months before data collection, whose emotional state may be in a period of abnormal fluctuation; II. Adolescents with severe mental illnesses (such as schizophrenia, severe depression, etc.) and cognitive impairments (such as intellectual disability, etc.); III. Adolescents whose native language is not Chinese or who lack a sufficient Chinese-speaking environment. In this study, screening questions corresponding to the inclusion and exclusion criteria were set in the “General Information Questionnaire, and combined with manual verification and logical validation after data collection to ensure that participants strictly met the preset criteria. Please refer to the **Supplementary Material** titled “01-General Information Questionnaire” for details.

### Sample size and power estimation

The sample size calculation in this study followed the rigorous logic of scale validation research and was determined step-by-step by integrating classical methods, statistical power analysis, and factor analysis standards. Firstly, the Kendall item multiple method was used for basic estimation. This method is based on the principle that “the sample size should be 5–10 times the number of items to stably detect the item-dimension association” (13). With 20 items in the initial scale, the basic sample size range was 100–200. Referring to the common 20% sample attrition rate in adolescent surveys (13), the sample size was adjusted using the formula “adjusted sample size = basic sample size ÷ (1–attrition rate)”, resulting in an initially planned sample size of 130–260. Secondly, *a priori* power validation was conducted using the “Correlation: Bivariate normal model” module in G*Power 3.1 software (suitable for item-dimension association testing). The parameters were set as follows: effect size r = 0.3 (14), Type I error rate α = 0.05, and statistical power 1–β= 0.8 (15a). A sample size of 84 was required for testing a single item. Since 19 final items needed to be tested, Bonferroni correction was applied (α = 0.05÷19 ≈ 0.0026), leading to an adjusted sample size of 160. Finally, considering the minimum standards for factor analysis (EFA ≥100, CFA ≥200 with item-to-sample size ratio ≥ 1:10) (16), 200 was determined as the required valid sample size. Adding a 20% attrition rate, the planned sample size for recruitment was 250. To further enhance the robustness and generalizability of the results, a total of 827 eligible adolescents were ultimately recruited via convenience sampling, and randomly divided into an EFA group (n = 527) and a CFA group (n = 300), which fully met the statistical requirements for scale reliability and validity analysis.

### Translation and cross-cultural adaptation

This study obtained authorization by contacting the original authors via email, and then translated the PEES-GAP into a Chinese version based on the modified Brislin model (17).

#### Scale introduction

PEES - GAP was developed by Professor Morenikeji Fausiat Hamzat’s team based on interdisciplinary theories. The scale consists of 20 items, including Hostility (Items 1 - 6), Critical Comments (Items 7 - 13) and Over - Involvement (Items 14 - 20). It adopts a 5 - point Likert scoring method, with response options ranging from 1 point (“Never”) to 5 points (“Always”). The total score ranges from 20 to 100 points, and a higher score indicates a higher level of expressed emotion in general adolescents. The original scale has good reliability and validity. Its reliability and validity were verified among 1,740 adolescents selected from six middle schools and three universities in three states in southwestern Nigeria. In terms of reliability, the scale demonstrated an overall internal consistency with a Cronbach’s α of 0.800. The α coefficients for all three core dimensions were above 0.4, and the item stability was satisfactory, ensuring consistent and reliable measurement results. Regarding validity, the construct validity analysis revealed that the three extracted factors collectively explained 37.16% of the total variance, with item loadings meeting the required standards. For convergent validity, the scale showed a significant positive correlation with the Index of Family Relations (IFR) (r = 0.523–0.718). For discriminant validity, it exhibited near-zero correlations with the Drug Abuse Screening Test (DAST-20) (r = -0.257 to -0.014). These findings fully confirm that the scale can accurately measure the target construct of perceived expressed emotion among adolescents (12).

#### Translation and back-translation of the scale

Step 1: Two postgraduate students majoring in psychology, whose native language is Chinese and with a CET-6 level of English proficiency, translated the PEES-GAP into two Chinese versions, namely T1 and T2. Subsequently, the first author integrated the two translated versions, conducted discussions and revisions, and finally developed the Chinese version T of the scale.
Step 2: Another psychology doctor and an English master whose native language is Chinese independently back-translated Scale T into English versions. Since they had no prior exposure to the scale, two English versions, NT1 and NT2, were generated in this process.
Step 3: The experts from Step 2 and the first author held a meeting to discuss versions NT1 and NT2. After confirming that there were no significant discrepancies with the original scale, the final back-translated version T3 was formed.

#### Cross-cultural adaptation of scales

In accordance with cultural adaptation guidelines, six experts were invited to evaluate the Chinese version of PEES-GAP through two rounds of email and on-site consultations. This process aimed to balance conceptual equivalence and cultural adaptation, ensuring that the language conforms to the linguistic norms of the region. Such a cultural adaptation approach is consistent with the original intention of the PEES-GAP, which was designed to be culturally sensitive to ensure relevance across diverse backgrounds.

Expert Basic Information and Authority Coefficient:The average age of the six experts was 46 ± 14 years, with detailed demographic data presented in **Table 1**. The calculation of the expert authority coefficient (Coefficient of Relative Authority, Cr) and its consultation results, the specific Likert scales for evaluation, the quantitative values of Coefficient of Judgment (Ca) and Coefficient of Familiarity (Cs), as well as the expert selection and two-round consultation process, are detailed in **Supplementary Material** titled “02-Expert Authority Coefficient”.

**Table 1:** Expert basic information.

Expert Number	Sex	Educational background	Professional title	Research direction	Years of professional experience	Age
A1	Male	Doctoral degree	Professor	Mental Health Education	22years	51 years old
A2	Female	Doctoral degree	Professor	Child and Adolescent Psychiatric Research	30 years	70 years old
A3	Female	Master’s degree	Associate Professor	Mental Health Education	19 years	48 years old
A4	Female	Master’s degree	Lecturer	Applied Psychology	13 years	39 years old
A5	Female	Master’s degree	Lecturer	Child and Adolescent Psychiatric Research	12 years	38 years old
A6	Female	Master’s degree	Senior Laboratory Technician	Mental Health Education	1 years	32 years old

### Measurement and instruments

1. General Information Scale: After reviewing relevant literature, the researchers designed a demographic data questionnaire by themselves to collect information such as age, sex, current school level, parents’ marital status, parents’ occupations, and grade ranking.
2. The Chinese version of the Perceived Expressed Emotion Scale for the General Adolescent Population (C-PEES - GAP) includes 19 items across three dimensions: Hostility (Items 1-6), Critical Comments (Items 7-13), and Over-Involvement (Items 14-19). It uses a 5-point Likert scoring method, with responses ranging from 1 point (“Never”) to 5 points (“Always”). The total score ranges from 19 to 95 points, and a higher score indicates a higher level of expressed emotion among general adolescents.

### Data collection

#### Pre-survey

In April 2025, 50 adolescents were selected as pre-survey subjects using the convenience sampling method in Jinzhou City, Liaoning Province, China. The participants had a mean age of 17.42 ± 1.279. Regarding sex distribution, there were 19 males (38%) and 31 females (62%). In terms of educational background, 2 participants had a junior high school education (4%), 10 had a senior high school education (20%), and 38 had a college education (76%).After understanding the purpose and significance of the study, all college students themselves and the guardians of middle school students signed the informed consent forms. The pre-survey feedback showed no semantic comprehension difficulties among participants, with an average questionnaire completion time of approximately 3 minutes. Additionally, participants generally recognized the scale as having a clear theme, complete structure, and coherent logic. Given these findings—particularly the smooth completion experience and positive evaluations of the scale’s design—no modifications were made, and the Chinese version of the PEES-GAP (C-PEES-GAP) was ultimately finalized.

#### Formal investigation

Before the formal survey, we contacted the head teachers and counselors of the adolescents, explained the purpose of the study to them, and provided guidelines for filling out the questionnaires. The survey instructions specifically emphasized that the data would be used exclusively for scientific research. Informed consent forms for parents were distributed to the guardians of middle school students through their head teachers, and all participants were informed that their participation was anonymous and voluntary. After obtaining support, we selected survey subjects who met the inclusion criteria and collected questionnaires in paper form. Participants were given uniform instructions, and after obtaining informed consent, they remained anonymous throughout the process, with the questionnaires ensured to be collected within 10 minutes. The data processing procedures and sample exclusion criteria of this study are as follows:

1. Data Processing Procedures: After questionnaire collection, questionnaires with incomplete responses, logical contradictions, or obviously random responses were first eliminated through manual verification. Incomplete responses refer to unanswered key items; logical contradictions refer to completely conflicting answers to items within the same dimension; obviously random responses refer to selecting the same option for all items. The initially screened questionnaire data were then entered into Excel 2021 for organization, and SPSS 25.0 was used to verify data integrity. After confirming no missing values, the data were used for subsequent reliability and validity analyses, including item analysis, EFA, and CFA.
2. Exclusion Criteria: In accordance with the pre-established participant inclusion and exclusion framework of the study, two types of objects were excluded. The first type was participants who did not meet the inclusion criteria, specifically adolescents who had experienced major life events within 3 months, suffered from severe mental illnesses or cognitive impairments, or were non-native Chinese speakers or lacked a Chinese-speaking environment. Major life events included the death of relatives and parental divorce; severe mental illnesses included schizophrenia; cognitive impairments included intellectual disabilities. The second type was questionnaires with invalid responses, specifically incomplete answers where key items are unanswered, logical contradictions with completely conflicting answers within the same dimension, and obvious random responses that select the same option for all items without genuine answering tendency. Finally, 73 invalid questionnaires were excluded from the 900 distributed questionnaires, and 827 valid questionnaires were retained, with an effective recovery rate of 91.85%. During the survey, participants voluntarily provided contact information for a reliability retest and could withdraw from the survey at any stage of the study. Two weeks later, 50 adolescents were selected from the initial participants to complete the same questionnaire to assess the test-retest reliability.

To ensure methodological rigor, avoid potential biases arising from using the same sample for both EFA and CFA, and ensure that the dimensions align with the expected ones, we randomly allocated the total sample (N = 827) into two independent subsamples. Among them, 527 adolescents were assigned to the EFA group, and the remaining 300 to the CFA group (16). This design allowed us to independently identify the factor structure in EFA and validate it through CFA, thereby effectively enhancing the psychometric robustness of the scale.

### Data analysis

Staff entered the questionnaire data into an Excel 2021 spreadsheet and used SPSS 25.0 and AMOS 26.0 software for data analysis. Count data were described using frequencies, percentages, or rates, and measurement data were described using means and standard deviations. Data were considered to be normally distributed when the skewness and kurtosis values of the items were between -2 and +2 (18). Before the formal analysis, we conducted an analysis of missing data to ensure data integrity and improve research transparency. Results obtained using SPSS25.0 showed that the dataset was complete with no missing values. Therefore, there was no need for imputation or other missing data processing techniques. Details of the data analysis software are provided in the **Supplementary Material** titled “03-Data Analysis Software”.

#### Item analysis

This study used the critical ratio method and correlation coefficient method to screen the items of the scale. (1) Critical ratio method: Independent sample t-test was conducted for the high-score group (top 27%) and low-score group (bottom 27%) to assess whether the differences were statistically significant. After sorting 827 questionnaires according to the total score, items with a critical ratio > 3 and statistical significance were retained (19). (2) Correlation coefficient method: Pearson correlation coefficient was used to assess the relationship between the 20 items and the total score. If the correlation between an item and the total score was less than 0.4, the item would be deleted (20). Since the 20th item “ I often get my relatives’ support whenever I need it” had a correlation with the total score of less than 0.4, we deleted this item. Finally, 19 items from the original scale were retained.

#### Validity analysis

Content Validity: Four psychological experts and two psychiatric experts were invited to evaluate the content validity of PEES - GAP using the Delphi method. A 4-point Likert scale was used for the evaluation, and each item was rated according to its relevance to the theme as follows: irrelevant = 1 point, weakly relevant = 2 points, relatively relevant = 3 points, and strongly relevant = 4 points. The I-CVI is calculated as the proportion of all experts who rated an item 3 or 4 points (21). The Average Scale Content Validity Index (S-CVI/Ave) is calculated by taking the average of all Item Content Validity Indices (I-CVI) (21). If the number of experts is 6 or more, it is required I-CVI is greater than 0.78, and S-CVI/Ave is 0.83 or higher (22).

Construct Validity: The potential factor structure of the translated scale was tested through EFA and CFA. In the EFA stage, principal component analysis was performed using the orthogonal rotation method (varimax). According to relevant studies (23), principal component analysis and varimax rotation are commonly used in EFA due to their ability to reduce cross-loadings and produce a clearer factor structure. Therefore, we adopted this method while examining eigenvalues and scree plot results. In the CFA stage, AMOS software was used to evaluate the fit indices of the model. In exploratory factor analysis (EFA), factors with an eigenvalue > 1 are selected, with factor loadings > |0.4| and each factor containing > 3 items—these criteria ensure the stability and interpretability of the factors (24). Based on the results of EFA, confirmatory factor analysis (CFA) is further conducted. Goodness-of-fit indices are used to test the model fit, including chi-square to degrees of freedom ratio (χ²/df), root mean square error of approximation (RMSEA), goodness of fit index (GFI), adjusted goodness of fit index (AGFI), comparative fit index (CFI), Tucker-Lewis index (TLI), and root mean square residual (RMR). The acceptable criteria for model fit are defined as follows: χ²/df < 3, CFI > 0.8, GFI > 0.8, IFI > 0.8, TLI > 0.8, AGFI > 0.8, and RMSEA < 0.08. The ideal criteria for model fit are: CFI > 0.9, GFI > 0.9, IFI > 0.9, TLI > 0.9, AGFI > 0.9, and RMSEA < 0.09 (24).

Convergent Validity and Discriminant Validity: Based on the results of CFA, the correlation coefficients between observed variables, Average Variance Extracted (AVE), and Critical ration(CR) were calculated. The composite reliability (CR) should exceed 0.6, and the average variance extracted (AVE) should be maintained above 0.5 (25). Discriminant validity was first tested using the Fornell-Larcker criterion. Notably, the results showed that the square root of AVE for each latent variable was smaller than the correlation coefficient between that latent variable and other latent variables. Finally, to demonstrate discriminant validity, we employed the multi-factor method by comparing the original model with models from which certain dimensions had been removed (26). The results confirmed that all indicators of the original model were superior to those of the other models, and it passed the significance test at the 0.01 significance level, thus verifying the discriminant validity of the model (**Table 2**).

**Table 2:** Discriminant and convergent validity of the C-PEES - GAP (n=300).

Discriminant validity					Convergent validity				
Factor	FA	FB	FC	Items	Sth.Estimate	SE	P	CR	AVE
FA		0.883**	0.908**	Ho1	0.726			0.9167	0.6487
	**0.805**								
				Ho2	0.842	0.073	P<0.001		
				Ho3	0.834	0.071	P<0.001		
				Ho4	0.869	0.066	P<0.001		
				Ho5	0.839	0.075	P<0.001		
				Ho6	0.708	0.094	P<0.001		
FB			0.829**	Cr1	0.777			0.9013	0.5663
		**0.752**							
				Cr2	0.754	0.081			
				Cr3	0.804	0.079	P<0.001		
				Cr4	0.741	0.076	P<0.001		
				Cr5	0.736	0.075	P<0.001		
				Cr6	0.714	0.083	P<0.001		
				Cr7	0.738	0.076	P<0.001		
FC				Ov1	0.741			0.5189	0.5189
			**0.702**						
				Ov2	0.756	0.052	P<0.001		
				Ov3	0.751	0.072	P<0.001		
				Ov4	0.885	0.074	P<0.001		
				Ov5	0.791	0.074	P<0.001		
				Ov6	0.140	0.113	P<0.001		

Bold text is the square root of AVE; **P<0.001.

#### Reliability analysis

This study evaluated the reliability of the measurement tool through test-retest reliability and internal consistency. To assess internal consistency, we calculated the Cronbach’s alpha coefficient for each dimension of the C-PEES - GAP scale. Fifty adolescents who voluntarily provided contact information in the first survey were randomly selected as the sample for test-retest reliability analysis, and the stability of the measurement tool was verified by calculating the correlation between the two sets of data. In addition, after dividing the scale items into two equal halves, the split-half reliability was evaluated by calculating the correlation between the two halves.

## Results

The C-PEES - GAP scale consists of 3 dimensions, namely Hostility, Critical Comments, and Over-Involvement, with a total of 19 items. Through item screening, Item 20th of the original scale, which has a correlation coefficient with the total score of < 0.4, was deleted.

### Participants

A total of 827 participants were included in the final study, with an age range of 14–19 years (17.85 ± 0.35). For more details, please refer to **Table 3**. In terms of age, the original scale selected adolescents aged 13–19 as the core sample. To ensure that the localized version aligns with the original scale in terms of the “emotional development characteristics of the measurement objects”, this study retains the core age range of 13–19 years old, so as to avoid the impact of differences in population age characteristics on the measurement equivalence of the localized scale (12).The classification of parental occupations is based on the Classification of Occupations in the People’s Republic of China (*National Occupational Classification Ceremony (2022 Edition) Public Notice*, n.d.).

**Table 3:** Distribution of demographic characteristics (N = 827).

Variables		Frequency	Percentage%
Sex	males	271	32.8
	females	556	67.2
Age	13-15	37	4.5
	15-17	189	22.8
	17-19	601	72.7
Education background	Junior high school	16	1.9
	Senior high school	214	25.9
	College	597	72.2
Grade Ranking	Top25%	334	40.4
	Top50%	286	34.6
	Top75%	155	18.7
	Bottom25%	52	6.3
Parents’ marital status	Married (including first marriage, remarriage, and remarriage with former spouse)	644	77.9
	Divorced	45	5.4
	Widowed	10	1.2
	unspecified	128	15.5
Parental occupation	Organizational Heads	97	11.7
	Clerical Staff	82	9.9
	Professional Technicians	164	19.8
	Service Workers	172	20.8
	Agricultural & Fishery Workers	121	14.6
	Manufacturing Workers	142	17.2
	Unemployed	49	5.9

### Cross-cultural adaptation

Considering Chinese cultural habits and combined with experts’ opinions, we decided to change “relatives” in the scale to “family” to better fit the habits and context in Chinese culture. In Chinese culture, the scope of “relatives” is too broad, and many teenagers find it difficult to understand why their relatives care about them so much, while the scope of “family” is just right, and care from family members will not make them confused or disgusted. At the same time, we also suggest changing “panic” in Item 16 to “extremely worried” because in Chinese culture, when teenagers are restless, their parents often show not panic but worry. Moreover, with the word “panic”, teenagers will also find it hard to understand why their parents should panic. The above adjustments were also affirmed by the interviewees in the pre-interviews. As for whether cultural adaptation will affect the conceptual equivalence between the adapted scale and the original version, we will conduct further research in future studies.

### Item analysis

A total of 20 items had critical ratio values ranging from 12.838 to 27.416 (all > 3, P < 0.001) (19). Pearson correlation analysis was used to examine the relationship between individual item scores and total scores, and the correlation coefficients r were found to range from 0.432 to 0.765 (all > 0.4, P < 0.001) (27), as shown in **Table 4**.

**Table 4:** Critical ratios of C-PEES - GAP, item-total correlation coefficients, and Cronbach’s alpha values after item deletion (n=827).

Item	Critical ratio	Correlation item total score	P	Cronbach’sα after deleting the item	Skewness/Kurtosis
Ho1	18.118	0.612	p<0.001	0.925	0.636/-0.553
Ho2	22.143	0.702	p<0.001	0.923	0.861/0.034
Ho3	24.456	0.727	p<0.001	0.922	1.076/0.367
Ho4	24.950	0.696	p<0.001	0.923	0.967/0.069
Ho5	25.231	0.732	p<0.001	0.922	0.883/-0.013
Ho6	27.416	0.714	p<0.001	0.923	0.613/-0.724
Cr1	19.584	0.671	p<0.001	0.924	0.543/-0.467
Cr2	21.146	0.660	p<0.001	0.924	0.726/-0.272
Cr3	21.679	0.689	p<0.001	0.923	0.551/-0.761
Cr4	20.318	0.656	p<0.001	0.924	0.616/-0.597
Cr5	20.015	0.676	p<0.001	0.924	0.727/-0.279
Cr6	22.154	0.712	p<0.001	0.923	0.683/-0.495
Cr7	20.198	0.689	p<0.001	0.923	0.899/0.180
Ov1	20.375	0.672	p<0.001	0.924	0.757/-0.383
Ov2	24.861	0.765	p<0.001	0.922	1.053/0.311
Ov3	23.285	0.671	p<0.001	0.924	0.857/0.230
Ov4	18.120	0.538	p<0.001	0.928	0.667/-0.741
Ov5	20.083	0.616	p<0.001	0.925	0.720/-0.496
Ov6	12.838	0.432	p<0.001	0.929	0.439/-0.859

### Validity

#### Content validity

This study invited six experts to evaluate the content validity of the C-PEES - GAP scale using the Delphi method. I-CVI and S-CVI/Ave were calculated based on a 4-point Likert scale. The results showed that the distribution of the Item-level Content Validity Index (I-CVI) for the 19 items in this study is as follows: 12 items had an I-CVI of 1.00, 7 items had an I-CVI ranging from ≥0.83 to <1.00 (≥ 0.78) (28), and the S-CVI/Ave value was 0.947 (≥ 0.83) (28).

#### Construct validity

**Figure 1:**
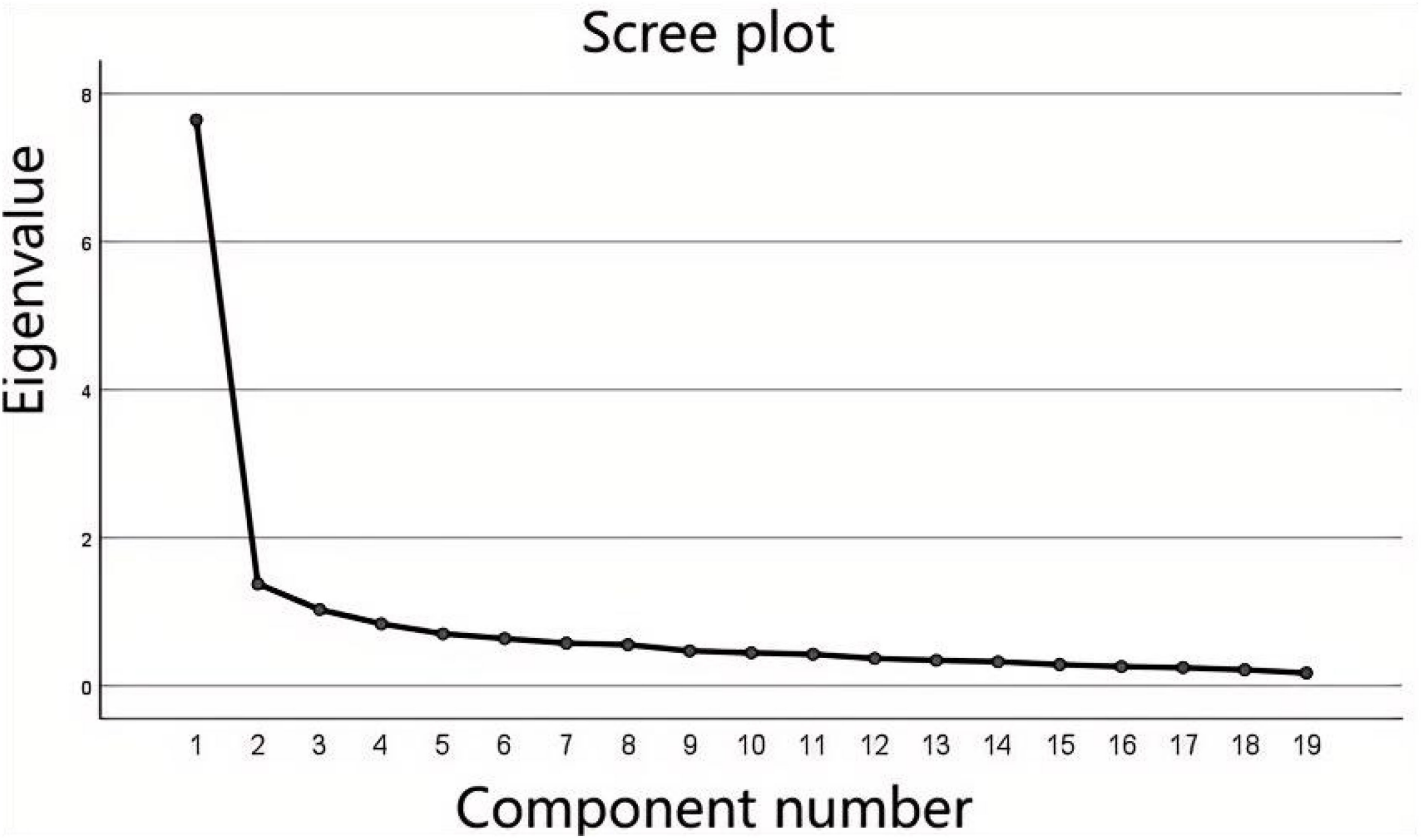
Scree plot for the C-PEES - GAP exploratory factor analysis(n = 527).

Exploratory factor analysis: Before conducting Exploratory Factor Analysis (EFA), we first performed the Kaiser-Meyer-Olkin (KMO) sampling adequacy test and Bartlett’s test of sphericity. It is generally considered that when the KMO value is > 0.7 and the P value is < 0.05 (29), the sample size is suitable for factor analysis. In this study, the KMO value was 0.941, and Bartlett’s test of sphericity yielded a chi-square value of approximately 5262.012 (degrees of freedom = 171, P < 0.05). Principal Component Analysis (PCA) was used to extract factors with eigenvalues > 1 (30). A component matrix was obtained through orthogonal varimax rotation, and only factors with loadings > 0.5 were retained (31) (**Table 5**). After 25 rotation iterations converged, a total of 3 indicators consistent with the original scale were extracted, with a cumulative explained variance of 59.295% (**Figure 1**). A cumulative explained variance of 59.295% exceeds the minimum psychometric standard of “cumulative explained variance ≥ 40%”, indicating that the scale has strong construct representativeness and excellent construct validity (32). It is much higher than the variance explanation rate of 37.161% of the original scale. This may be because, during the localization process, certain items were semantically adjusted to align with the Chinese cultural context, thereby enhancing their relevance to the language habits and cognitive styles of the target population. This likely improved the consistency of participants’ interpretations of the items. No items had a loading > 0.40 on non-target factors, indicating no cross-loading issues, and thus no items need to be deleted or adjusted.

**Table 5:** Factor loadings of exploratory factor analysis for the C-PEES - GAP (n=527).

Item	FactorA	FactorB	FactorC
Ho1			0.703
Ho2			0.751
Ho3			0.655
Ho4			0.614
Ho5			0.580
Ho6			0.592
Cr1	0.621		
Cr2	0.621		
Cr3	0.729		
Cr4	0.734		
Cr5	0.760		
Cr6	0.673		
Cr7	0.674		
Ov1		0.669	
Ov2		0.708	
Ov3		0.740	
Ov4		0.669	
Ov5		0.714	
Ov6		0.571	

**Figure 2:**
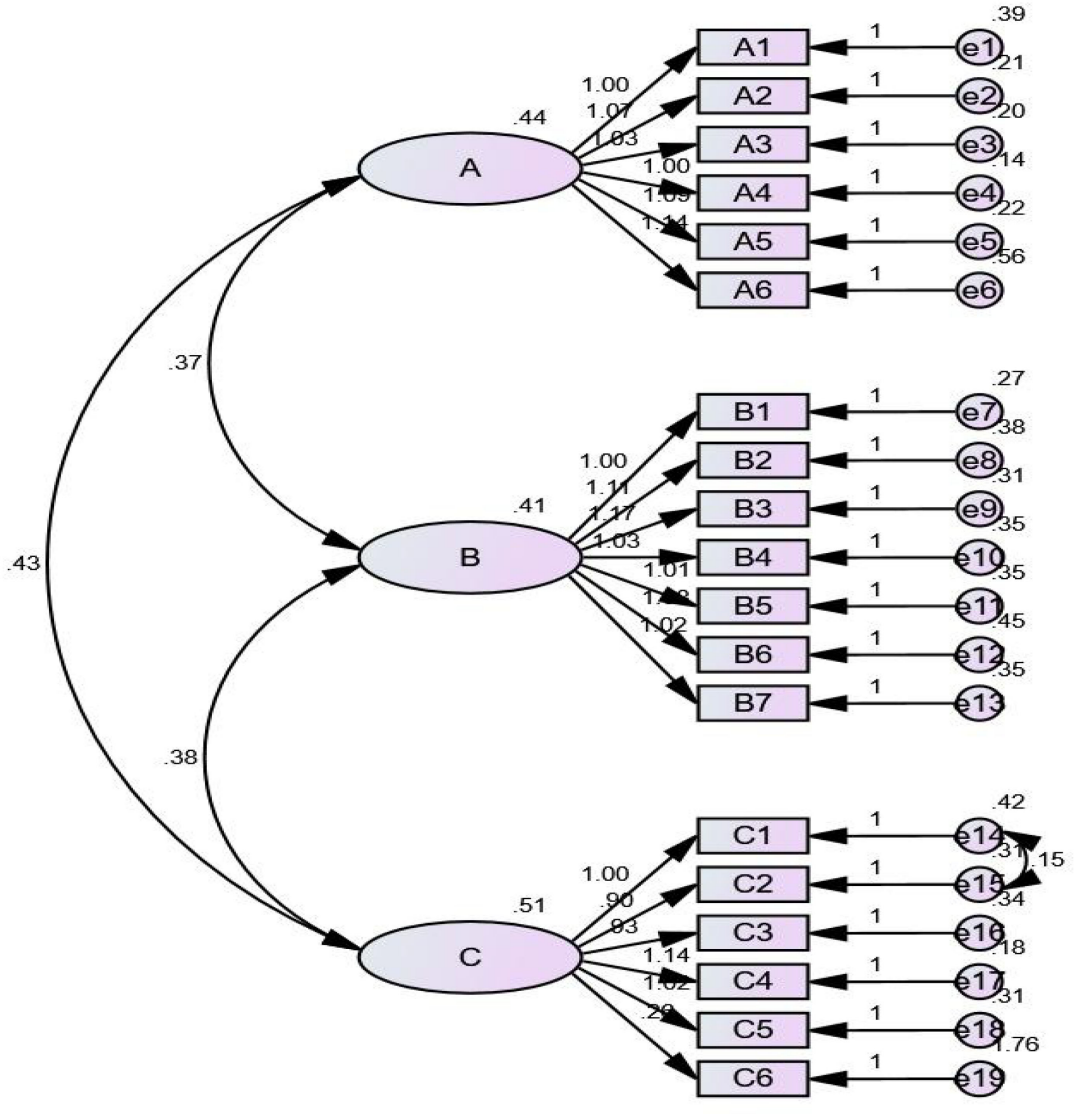
Hypothesized confirmatory factor analysis model of the C-PEES - GAP (n=300). **(A)** Hostility **(B)** Critical comments **(C)** Over involement.

Confirmatory factor analysis: CFA aims to test whether the relationship between questionnaire items and factors conforms to preset assumptions. Model fit indicators include: Chi-square Goodness of Fit (CMIN/DF), Root-mean-square error of approximation (RMSEA), Adjusted goodness-of-fit index (AGFI), Goodness-of-fit index(GFI), Tucker lewis index (TLI), Incremental fit index (IFI), Comparative fit index(CFI), and Parsimony goodness-of-fit index (PGFI). The initial model did not meet the expected standards. According to the Modification Index (MI) (33), the initial model adjusted e14 and e15 by correlating error terms (**Figure 2**) The adjustment to correlate the error terms of e14 (“I feel overly protected by my family”) and e15 (“My family’s overprotection makes me feel like I don’t even know myself”) is supported by both theoretical and statistical evidence. Theoretically, both items belong to the “Over-Involvement” dimension and centrally revolve around “family overprotection”: the former reflects the direct perception of this behavior, while the latter describes the impact of this behavior on self-cognition. Together, they form a “behavior-result” logical association, thereby creating shared error variance caused by content homology—a common phenomenon in scale design where items with closely related concepts often have unmodeled shared variance (34). After correlating the error terms, the fit indices of the revised model (M2) improved significantly: the chi-square to degrees of freedom ratio (CMIN/DF) decreased to 2.791, the root mean square error of approximation (RMSEA) dropped to 0.077, and indices such as the Tucker-Lewis Index (TLI) and Comparative Fit Index (CFI) all met ideal standards. Meanwhile, item factor loadings remained stable (> 0.5), indicating that the core structure of the “Over-Involvement” dimension was not disrupted (35). However, this adjustment has an inherent limitation of sample dependence—its effectiveness may be influenced by the cognitive characteristics of the adolescent sample in this study regarding “overprotection”. **Table 6** shows the final model fit indicators: CMIN/DF = 2.791 (< 3), RMSEA = 0.077 (< 0.08), AGFI = 0.832, TLI = 0.924, IFI = 0.935, CFI = 0.934, GFI = 0.869, and PGFI = 0.677 (> 0.5) (25). **Table 2** shows that the CR ranges from 0.8527 to 0.9167 (> 0.7), while the AVE ranges from 0.5189 to 0.6487 (> 0.5) (19). Although AGFI and GFI do not reach the ideal threshold of the model, they are still within the acceptable range since GFI > 0.85 and AGFI > 0.8 (36). Theoretically, this may raise concerns about the “structural representativeness” of the C-PEES-GAP scale. However, other key indicators of Confirmatory Factor Analysis (CFA) all meet the ideal standards; moreover, in the Exploratory Factor Analysis (EFA), the cumulative variance explained by the three factors reaches 59.295%, which is much higher than the 37.16% cumulative variance explained by the original PEES-GAP scale. These results fully indicate that the construct validity of the C-PEES-GAP scale in this study was not substantially affected by the relatively low values of AGFI and GFI. Although the standardized factor loading of item Ov6 in **Table 6** was far below the recommended threshold of 0.50 during the confirmatory factor analysis (CFA), its inherent meaning (“I am often asked many personal questions by my family members”) holds significant importance for the Over involvement dimension (37). Furthermore, this item exhibited a favorable factor loading in the previous exploratory factor analysis (EFA). This discrepancy in loading values is likely attributed to issues related to sample size, and we will conduct further verification in subsequent studies.

**Table 6:** Model fit indices of C-PEES - GAP before and after modification in confirmatory factor analysis.

Model	CMIN/DF	RMSEA	AGFI	TLI	IFI	CFI	GFI	PGFI
M1	3.069	0.083	0.822	0.912	0.924	0.923	0.860	0.674
M2	2.791	0.077	0.832	0.924	0.935	0.934	0.869	0.677
Standard	<3	<0.08	>0.9	>0.9	>0.9	>0.9	>0.9	>0.5
Model fit interpretation	good	good	acceptable	good	good	good	acceptable	good

M1: Before Modification; M2: After Modification.

### Reliability

The overall Cronbach’s alpha coefficient of the C-PEES - GAP scale is 0.928. The Cronbach’s alpha coefficients of the three factors are 0.858, 0.878, and 0.827 (38), all exceeding the threshold of 0.7. In addition, the test-retest reliability after a two-week interval is 0.964(>0.7) (39), with a 95% confidence interval of [0.937, 0.980]. When calculating the split-half reliability, the first-half *vs*. second-half splitting method and the Spearman-Brown formula were adopted, yielding a value of 0.855 (>0.7)(19), both of which meet the requirements of the established psychometric standards. Among them, 50 participants were selected for the test-retest reliability assessment, with a mean age of 16 ± 1.480. Regarding sex distribution: 38 were female (64%) and 18 were male (36%). In terms of educational background: 7 had a junior high school education (14%), 36 had a senior high school education (72%), and 7 had a college education (14%). A two-week interval is chosen for test-retest reliability to avoid the memory effect of an excessively short interval—like within a few days—since this stops participants from recalling first-measurement responses and undermining the second result’s independence (15). It also prevents issues from an overly long interval, such as several months, which would cause measured traits (e.g., psychological traits, behaviors) to change significantly over time or be disrupted by external factors, failing to reflect the scale’s true stability (15).

## Discussion

### C-PEES - GAP has appropriate discrimination

C-PEES - GAP demonstrates good performance in quantitative research. The core of item analysis lies in evaluating the discriminative effectiveness of the scale and each item. To reduce potential biases introduced by a single assessment method, this study jointly adopted the critical ratio method and correlation coefficient method for item screening. Using an independent samples t-test to compare score differences between the high-score and low-score groups, all items yielded t-values that met the discriminative criterion (t > 3.0, all P < 0.001), indicating significant discriminative power for each item (40). Pearson correlation analysis was conducted to examine the correlation between each item and the total scale score. Except for the initially translated Item 20 (r < 0.4, not meeting the standard), the remaining 19 items all showed correlation coefficients that met the validity criterion (r > 0.4, all P < 0.001). This confirms a significant correlation between each qualified item and the scale as a whole, verifying the measurement validity of the scale (20).

In addition, the Cronbach’s alpha coefficient of the revised C-PEES-GAP scale was 0.928. Although the Cronbach’s alpha coefficient of the scale increased to 0.929 after removing Item 19 from the revised scale, according to the criteria proposed by Wang Hanyi, an item should only be considered for removal if its deletion results in an increase in the Cronbach’s alpha coefficient by more than 0.5 (41). Since the deletion of Item 19 did not have a substantial impact on the reliability of the remaining items, it was ultimately retained.

In summary, C-PEES - GAP has deleted Item 20 of the original scale and retained 19 items, which has a high degree of homogeneity and strong discriminative power.

### C-PEES - GAP has appropriate validity

Content validity assessment aims to verify whether the scale items accurately reflect the construct being measured. Domain experts will conduct a comprehensive assessment of the scale content to ensure its applicability. This study invited six experts to conduct content validity assessment and cultural adaptation adjustment of the scale. The results showed that the reliability index S-CVI/Ave of the translated scale was 0.947, indicating good overall content validity (22).

Differences in domain-specific expertise or professional experience may result in inconsistencies in the scoring of certain items. However, the I-CVI values range from 0.83 to 1.00, which exceed the recommended threshold (22), and this supports the content validity of the translated version. The above results indicate that the C-PEES - GAP scale has been highly recognized by professionals. Its linguistic expression is in line with the Chinese cultural background and language norms, and it is highly understandable.

Construct validity, as a theoretical-level validity indicator reflecting the conceptual framework of the research object, was explored in this study through EFA with varimax rotation, which identified 3 latent factors, named “Hostility”, “Critical Comments”, and “Over-Involvement” respectively. These factors are highly consistent with the corresponding dimensions of the English version of the scale. Moreover, the rotated factor loadings of all items are greater than 0.5, with no cross-loading phenomenon, fully meeting psychometric standards. The results of the construct validity analysis show that the cumulative variance explanation rate of the Chinese version of the scale is 59.295%, which is significantly higher than the 37.161% reported for the original version. This difference may stem from the factor: during the localization process, some items were semantically adjusted to make them more in line with the Chinese cultural context, as well as the language habits and cognitive patterns of the target population, thereby improving the internal consistency of the scale. In summary, the relatively high cumulative variance explanation rate indicates that this Chinese version of the scale has good construct validity.

In addition, the results of CFA show that the model’s key fit indices, including CMIN/DF, RMSEA, TLI, IFI, CFI, and PGFI, all meet the ideal criteria for model fit. Although the AGFI and GFI do not reach the ideal threshold of 0.9, they are still within the acceptable range (36). This deviation may be related to sample size limitations. Overall, the remaining fit indicators all meet the ideal standards, and the model fits well, which further confirms that the scale has strong construct validity.

Convergent validity is used to evaluate whether items measuring the same latent construct are reasonably grouped. The C-PEES - GAP scale shows excellent convergent validity across all three factors, with its CR and AVE values all meeting the ideal criteria for convergent validity.

Discriminant validity was first tested using the Fornell-Larcker criterion, but the results were not ideal. This may be due to excessive sample homogeneity, such as an overly limited number of regions from which the adolescents were recruited. Another possible reason is cultural differences in emotional expression: Western cultures encourage direct emotional expression (e.g., “It is normal to express anger”), while East Asian cultures (including Chinese culture) place greater emphasis on emotional restraint (e.g., “Forbearance is a sign of maturity”). Since the items of the original scale were designed based on Western patterns of emotional expression, such emotional restraint might cause adolescents to suppress their own emotional expression after the scale was localized into Chinese, leading to blurred boundaries between dimensions and an increase in the correlation coefficients between them. Finally, we verified the discriminant validity of the scale using the multi-factor method (26) (**Table 7**).

**Table 7:** Comparison of discriminant validity results.

Number	Model	CMIN	DF	CMIN/DF	RMSEA	TLI	IFI	CFI	Model comparison	△CMIN	△DF
1	Original model	457.244	149	3.069	0.083	0.912	0.924	0.923			
2	Two factor model 1	570.397	151	3.777	0.096	0.882	0.896	0.896	2*vs*1	113.153**	2
3	Two factor model 2	617.067	151		0.102	0.869	0.885	0.884	3*vs*1	159.823**	2
4	Signal factor model	671.967	152	4.421	0.107	0.855	0.871	0.871	4*vs*1	214.723**	3

**representing p < 0.01.

Two factor model 1: FA+FB,FC.

Two factor model 2: FA, FB+FC.

Signal factor model: FA+FB+FC.

### C-PEES - GAP has appropriate reliability

Internal consistency reliability reflects the degree of consistency among all test items. A Cronbach’s alpha coefficient of less than 0.6 indicates insufficient internal consistency; a reliability coefficient between 0.7 and 0.8 suggests moderate reliability; and a Cronbach’s alpha coefficient ranging from 0.8 to 0.9 denotes good reliability (38). In this study, the Cronbach’s alpha coefficient of the total score scale reached 0.928, which was significantly higher than that of the original scale (0.79). This improvement is mainly attributed to the accurate refinement and optimization of content during the localization process. By adopting a rigorous three-step method of “translation-back translation-expert revision”, the study adjusted some semantic expressions while retaining the original meaning, making the questions easier for participants to understand and answer accurately. In addition, the Cronbach’s alpha coefficients of each dimension ranged from 0.827 to 0.878, indicating extremely strong internal consistency among the 19 items of the translated version. Test-retest reliability is used to evaluate the stability of the scale results over time, with its correlation coefficient ranging from 0 to 1. The closer the value is to 1, the higher the reliability. The overall test-retest reliability in this study was 0.964, and the reliability of each dimension ranged from 0.883 to 0.971, showing good stability and consistency. The split-half reliability method assesses internal consistency by dividing the questionnaire items into two parts, which are regarded as two independently measured dimensions in a short period. The correlation coefficient between the two parts is the split-half reliability indicator. When the Spearman correlation coefficient is ≥ 0.7, it indicates good split-half reliability. The split-half reliability of the translated scale in this study was 0.855, showing good reliability (19).

With its good reliability and validity, the C-PEES - GAP scale combines cultural adaptability with the emotional and psychological characteristics expressed by adolescents. It has stronger pertinence, scientificity and rigor in evaluating the emotional psychology expressed by adolescents. Moreover, it is more in line with the living situations and emotional expression habits of Chinese adolescents, which improves the cultural applicability of the scale. It provides a reliable tool for psychological and psychiatric research related to emotional expression of Chinese adolescents.

## Limitations

First of all, convenience sampling was used to select 827 adolescents from two middle schools and two universities in Qingdao City, Shandong Province, and Jinzhou City, Liaoning Province as the research subjects. This sampling method may cause selection bias, limit the representativeness of the sample, and affect the generalizability of the results. To improve the generalizability of the research results, it is recommended to adopt random sampling or expand the sample to middle schools and universities in multiple provinces. Secondly, there were limitations in the expert evaluation for content validity: the study invited only six experts (including four psychological experts and two psychiatric experts) to assess the scale’s content validity. Although the expert authority coefficient (Cr) met the validity criterion (Cr≥0.7) and the I-CVI/S-CVI/Ave reached the acceptable standard, the relatively small number of experts may limit the comprehensiveness of the evaluation—for instance, experts with backgrounds in adolescent education or cross-cultural psychology were not included, which might have led to potential omissions in judging the scale’s adaptability to adolescents’ cognitive characteristics and cultural context. Thirdly, there may be sampling bias and confounding bias in this study. For example, factors such as adolescents’ age, sex, and family situation may affect their understanding of the scale and answering tendencies, thereby having a potential impact on the research results. Future studies can try to use stratified sampling (e.g., stratifying by age, school type, or region) or adjust statistical analysis methods (such as controlling potential confounding factors through multiple regression analysis) to improve the internal validity of the research. Finally, as there were no other scales for measuring expressed emotions from the perspective of ordinary adolescents, this study did not conduct validity correlation analysis. Despite these limitations, this study still followed strict procedures for translation, cultural adaptation, and reliability and validity verification to ensure the applicability and measurement quality of the scale. Future studies can further verify the applicability of this scale in different regions and populations, expand the expert team with diverse professional backgrounds for content validity evaluation, and adopt longitudinal research methods to test its long-term stability.

## Conclusion

This study strictly followed the modified Brislin translation model and successfully introduced the PEES-GAP scale, which has demonstrated strong reliability and validity in the Chinese cultural context. Furthermore, during the Sinicization process, the wording of items was optimized in combination with the family cultural background of Chinese adolescents to ensure that the language is easy to understand and conforms to the local context. In summary, the C-PEES-GAP can serve as a reliable tool for assessing expressed emotion from the perspective of adolescents, facilitating research and practice in adolescent mental health, and filling the gap in the applicability of existing scales.

## Data Availability

The original contributions presented in the study are included in the article/**Supplementary Material**. Further inquiries can be directed to the corresponding author.
