# Supplementary material for: Cross-cultural adaptation and validation of the Chinese version of the Perceived Emotional Expression Scale for adolescents
Source: Front Psychiatry. 2025 Dec 9;16:1680435. doi: 10.3389/fpsyt.2025.1680435 (PMC12722932; doi:10.3389/fpsyt.2025.1680435)
Supplement: Supplementary file 1 [file Table1.docx]

# General Information Questionnaire

This questionnaire is used to collect your basic information for research screening and data analysis. Please answer truthfully according to your actual situation.

## 1. Basic Demographic Information

1.1 Sex

- □ Males
- □ Females

1.2 Age

- □ 13-15 years old
- □ 15-17 years old
- □ 17-19 years old

1.3 Education Background

- □ Junior high school
- □ Senior high school
- □ College

1.4 Grade Ranking (in your current class/grade)

- □ Top 25%
- □ Top 50% (26%-50%)
- □ Top 75% (51%-75%)
- □ Bottom 25% (76%-100%)

## 2. Family-related Information

2.1 Parents' Marital Status

- □ Married (including first marriage, remarriage, remarriage with former spouse)
- □ Divorced
- □ Widowed
- □ Unspecified

2.2 Parental Occupation (select the main occupation of one or both parents, as applicable)

- □ Organizational Heads
- □ Clerical Staff
- □ Professional Technicians
- □ Service Workers
- □ Agricultural & Fishery Workers
- □ Manufacturing Workers
- □ Unemployed

## 3. Research Screening Questions

3.1 Have you experienced major life events (e.g., death of a relative, parental divorce) in the 3 months before completing this questionnaire?

- □ Yes
- □ No

3.2 Do you have a diagnosis of severe mental illnesses (e.g., schizophrenia, severe depression) or cognitive impairments (e.g., intellectual disability)?

- □ Yes
- □ No

3.3 Is your native language Chinese, and do you live in a sufficient Chinese-speaking environment?

- □ Yes
- □ No

Note: Your answers to this questionnaire will be kept strictly confidential and used only for this research. Thank you for your cooperation!
