# Supplementary material for: Cross-cultural adaptation and validation of the Chinese version of the Perceived Emotional Expression Scale for adolescents
Source: Front Psychiatry. 2025 Dec 9;16:1680435. doi: 10.3389/fpsyt.2025.1680435 (PMC12722932; doi:10.3389/fpsyt.2025.1680435)
Supplement: Supplementary file 2 [file Table2.docx]

**Expert Authority Coefficient**

1. The expert authority coefficient (Cr) is determined jointly by two factors: the experts’ judgment basis (Ca) for the indicators and their familiarity level (Cs), following the calculation formula: Cr = (Cs + Ca) / 2. Specifically, Ca (judgment basis) comprises four dimensions—theoretical analysis, practical experience, literature reference, and personal intuition—each assigned distinct weights based on its degree of influence on the evaluation. Cs is categorized according to how well experts understand the indicators, with each category corresponding to a specific numerical value. This calculation approach incorporates both the experts’ professional judgment capabilities and their cognitive understanding, ensuring the objectivity and reliability of the assessment(‘Endoscopic Supply Chain Management and Optimization’, 2021). The authority coefficients for the two rounds of expert consultation were Cr=0.78 (first round) and Cr=0.90 (second round), both meeting the validity criterion of Cr≥0.7 for expert consultation(J. Li, 2025). This indicates that the participating experts had a high level of authority, with further details provided in Table 1.3.
2. Specific Evaluation Methods：For evaluating the relevance of scale items, experts adopted a 4-point Likert scale, with the scoring standards defined as follows: 1 point for "irrelevant", 2 points for "weakly relevant", 3 points for "relatively relevant", and 4 points for "strongly relevant". A 5-point Likert scale (extremely high/high/medium/low/extremely low) was used to assess the influence degree of the judgment basis. The specific quantitative values corresponding to Ca and Cs are listed in Table 1.1 and Table 1.2, respectively.
3. Detailed Evaluation Procedures：Six experts specializing in related fields (including mental health education and applied psychology) were selected, with their professional experience ranging from 1 to 30 years. In the first round of consultation, questionnaires were sent to experts via email to collect their evaluations on the relevance of scale items, the judgment basis, and their modification suggestions. After optimizing the scale based on the feedback from the first round, the second round of consultation was carried out to further align experts’ opinions. To verify the validity of the evaluation results and the consistency of experts’ opinions, the item-level content validity index (I-CVI) and the Average Scale Content Validity Index (S-CVI/Ave) were calculated.

**Table 1.1** Quantitative standards for evaluation criteria (Ca)

| **Basis for judgment** | **Extremely high** | **High** | **medium** | **low** | **very low** |
| --- | --- | --- | --- | --- | --- |
| Theoretical analysis | 0.25 | 0.2 | 0.15 | 0.1 | 0.05 |
| Practical experience | 0.5 | 0.4 | 0.3 | 0.2 | 0.1 |
| Literature reference | 0.25 | 0.2 | 0.15 | 0.1 | 0.05 |
| Personal intuition | 0.15 | 0.15 | 0.1 | 0.05 | 0.05 |

**Table1.2** Experts' understanding of evaluation indicators (Cs)

| **Level of understanding** | **Cs** |
| --- | --- |
| Very familiar | 0.9 |
| Familiar | 0.7 |
| Somewhat familiar | 0.5 |
| Unfamiliar | 0.3 |
| Completely unfamiliar | 0.1 |

**Table1.3** Expert Authority Coefficient（Cr）

| **Project** | **Basis for judgment** | **Level of understanding** | **Authority coefficient** |
| --- | --- | --- | --- |
| First round | 0.86 | 0.7 | 0.78 |
| second round | 0.92 | 0.88 | 0.9 |
